# Underdiagnosis of Foodborne Hepatitis A, the Netherlands, 2008–2010^1^

**DOI:** 10.3201/eid2004.130753

**Published:** 2014-04

**Authors:** Mariska Petrignani, Linda Verhoef, Harry Vennema, Rianne van Hunen, Dominique Baas, Jim E. van Steenbergen, Marion P.G. Koopmans

**Affiliations:** Municipal Health Service Zoetermeer, Zoetermeer, the Netherlands (M. Petrignani, R. van Hunen);; National Institute for Public Health and the Environment (RIVM), Bilthoven, the Netherlands (L. Verhoef, H. Vennema, D. Baas, J.E. van Steenbergen, M.P.G. Koopmans);; Erasmus Medical Center, Rotterdam, the Netherlands (M.P.G. Koopmans);; Leiden Medical University, Leiden, the Netherlands (J.E. van Steenbergen)

**Keywords:** foodborne diseases, hepatitis A virus, epidemiology, molecular typing, public health, the Netherlands, enhanced surveillance, viruses

## Abstract

Outbreaks of foodborne hepatitis A are rarely recognized as such. Detection of these infections is challenging because of the infection’s long incubation period and patients’ recall bias. Nevertheless, the complex food market might lead to reemergence of hepatitis A virus outside of disease-endemic areas. To assess the role of food as a source of infection, we combined routine surveillance with real-time strain sequencing in the Netherlands during 2008–2010. Virus RNA from serum of 248 (59%) of 421 reported case-patients could be sequenced. Without typing, foodborne transmission was suspected for only 4% of reported case-patients. With typing, foodborne transmission increased to being the most probable source of infection for 16%. We recommend routine implementation of an enhanced surveillance system that includes prompt forwarding and typing of hepatitis A virus RNA isolated from serum, standard use of questionnaires, data sharing, and centralized interpretation of data.

Hepatitis A virus (HAV) infection is an acute, usually self-limiting, illness; transmission is associated with suboptimal hygiene. Transmission occurs by the oral route, and infected persons can shed high amounts of infectious virus in their feces (*1*). Over recent decades, the incidence of HAV infections has been declining to a low level of transmission in high-income and middle-income countries. This epidemiologic shift results in a gradual shift in patient age and severity of first infection, from asymptomatic infections in very young children toward more severe illness in older children and adults. The World Health Organization estimates a case-fatality rate ranging from 0.1% for children <15 years of age to 2.1% for adults >40 years of age (*2*). As incidence of HAV decreases, the proportion of the population vulnerable to infection increases. Thus, paradoxically, hepatitis A virus could reemerge in regions where it is not endemic, affecting mostly adults. Risk for outbreaks with more severe illness becomes greater in countries where such epidemiologic transition has occurred.

In countries with low levels of HAV, the main risk comes from travel, secondary waves of transmission in households and schools, and (ongoing and sometimes epidemic) transmission among men who have sex with men (MSM) (*3*–*11*). However, the probable source of infection remains unknown for 20%–30% of cases, possibly because of transmission by persons with subclinical or missed primary cases, but alternatively because of food contamination. Although HAV is listed as the second most common foodborne virus (*12*), foodborne HAV infections are rarely reported, except when triggered by an unusual outbreak or event. In general, detection of a food source is difficult because the incubation period for hepatitis A is long (average 4 weeks); therefore, responses to food-consumption questionnaires, if administered, might be unreliable because of recall bias. Moreover, the food industry is a complex multinational system, and many high-risk products (shellfish, fresh or frozen fruits and vegetables) are produced in HAV-endemic countries. The common methods used for microbiological quality control of food do not reliably predict presence or absence of virus contamination (*13*). Virus contamination of high-risk foods is not uncommon; some of these products have a long shelf life as frozen or dried products in which HAV can survive for at least 2–3 months (*14*,*15*), and these products can be marketed over a wide geographic region. For these reasons, foodborne HAV infections are difficult to recognize.

These surveillance challenges might discourage physicians from trying to signal foodborne outbreaks. Large outbreaks are detected because of their large numbers. Slow and dispersed clusters can be detected through use of molecular typing, which enables linking of cases that otherwise could not be recognized as a cluster (*16*,*17*).

We assessed the role of food as a source of HAV in the Netherlands, a country with low-level endemic circulation of HAV. To do so, we conducted a 2-year study in which we combined detailed epidemiologic investigation with real-time strain sequencing for reported case-patients.

## Methods

### Routine Surveillance

In the Netherlands, HAV infection is a reportable disease. Physicians and medical laboratories report cases to a municipal health service (MHS) according to national notification criteria: presence of a predefined set of clinical signs of hepatitis combined with HAV IgM in serum. MHS Consultants for Communicable Disease Control contact the patient and administer a questionnaire that collects routine demographic and epidemiologic data consisting of age, sex, country of birth, time of disease onset, related cases, travel history, homosexual contacts, and other possible modes of transmission (full questionnaire available on request to M.P.). MHS enters the suspected transmission route and other anonymized information into a national electronic registration system hosted by the National Institute for Public Health and the Environment (RIVM). All cases reported during July 1, 2008, through June 30, 2010, were included in the study.

### Enhanced Surveillance

During the 2-year period, an enhanced surveillance system, which included systematic typing of viruses from patients, was deployed. All medical microbiological laboratories and MHSs in the Netherlands were asked to send serum samples from all reported patients to the Laboratory for Infectious Diseases Research, Diagnostics and Screening at RIVM. RNA was extracted from the serum and tested for HAV by reverse transcription PCR selective for the viral protein (VP) 1–2A region of the genome (*3*,*4*). HAV genotyping was conducted by sequencing of a 460-nt fragment of the VP1–2A region. Sequence data were stored in a Bionumerics database (Sint-Martens-Latem, Belgium). Sequencing results were merged with the national registration data, according to laboratory name and serum sample number. For cases lacking a unique serum sample number, notification data and sequences were linked by using combinations of variables to match records (birth year, 4-digit postal code, date of illness onset, date of diagnosis).

The reporting MHSs were contacted twice by telephone for interviews. We asked for the MHS conclusion as to the most probable modes of transmission immediately after the notification and then after results from sequencing were available. This approach was taken because public health measures for different transmission categories might differ (Table 1) and interventions could be adjusted accordingly. The initial interviews were also used to inform MHS about the study and to emphasize the need for collection of serum samples. The conclusions as to possible mode of transmission before and after inclusion of typing information were logged separately.

**Table 1:** Hepatitis A virus transmission categories and supplementary public health actions, the Netherlands, 2008–2010

Category	Description	Public health action*
Travel-associated	History of travel to a country with high, intermediate, or low HAV endemicity (*18*)	Advise on future travel precautions
Person-to-person	Local contact with HAV-infected person	Widen contact tracing to identify risk groups and vaccination (e.g., school, health care setting, homeless, travel group)
MSM	Male-with-male sex	Widen contact tracing
Foodborne	Suspected food product or food handler	Trace sources (notify the food safety authority)
Unknown	No other applicable category	No further action

*In addition to hygiene measures, vaccination of household contacts, and restriction from school or work according to national guidelines; HAV, hepatitis A virus; MSM, men who have sex with men.

Because we used serum already available for diagnostic purposes, ethics approval was not needed. Patients were approached according to existing guidelines, and analyses maintained patient anonymity.

### Sequence Analyses and Strain Comparisons

The Bionumerics database already contained patient data and strain sequences from previous studies conducted in the Netherlands (*3*–*6*) and all available sequences from GenBank (*19*). These data were used for background comparison if sequences covered a minimum of 300 nt of the VP1–2a region and if information was available on the most probable country of infection (for travelers) or other risk activities (*20*,*21*). The geographic fingerprints and other risk-group associations (e.g., Dutch MSM strains) from the background data were used to classify strain sequences from patients with unknown exposure to a probable source. This association was reported to the MHS only if the association was considered robust; robust clusters consisted of at least 3 identical sequences from independent patients with the same country of infection or MSM association, branching separately in a maximum-parsimony tree with >75% reproducibility of bootstraps. Clusters were defined when the following were found: at least 2 identical sequences branching separately in a maximum-parsimony tree with >75% reproducibility of bootstraps. Maximum-parsimony trees (phylogenetic trees based on finding the simplest or minimal evolutionary change between strains) were built by using Bionumerics, and reproducibility was tested by performing 1,000 bootstraps. Cases with strains meeting this cluster definition and sharing the same suspected mode of transmission were considered confirmed clusters within the assigned transmission category.

### Descriptive and Statistical Analyses

Data analyses were performed by using SAS software version 9.2/9.3 (SAS Institute, Cary, NC, USA). We described the study population by age and sex, disease incidence, and the number and percentage of patients for whom the virus could be typed. We analyzed the representativeness of age distribution for patients for whom sequencing was performed. If date of onset of disease was unknown, we used the date of diagnosis as a proxy. We compared age distribution and, when available, lag time between onset of disease and PCR diagnosis of positive and negative cases to weigh a negative result. We described the number and percentage of most probable modes of transmission in 5 categories (Table 1) before and after inclusion of typing results.

## Results

A total of 421 cases were reported. Of these, serum samples could be obtained from 292 (69%) patients; HAV RNA from 248 (59%) of these samples could be typed.

### Description of Cases

The 421 cases reported over the 2-year period resulted in incidence rates of 1.2 cases/100,000 population during the study year 2008–09 and 1.3 cases during 2009–10 among a total population of 16.4 million at the start of the study period and 16.5 million at the start of the second year. Most reported patients were in age groups from 0–9 through 40–49 years (range 13.3%–18.8% per group; Table 2). For patients in the youngest age group (0–9 years), sequenced cases were underrepresented, although distributions for patients for whom sequencing was performed did not differ significantly from reported patients (data not shown).

**Table 2:** Description of reported patients with hepatitis A virus infection, by age group, the Netherlands, 2008–2010

Age group, y	Reported	Male, no. (%)	Incidence 2008–09*	Incidence 2009–10*	Sequenced, no. (%)†
0–9	78	41 (52.6)	1.6	2.4	29 (37.2)
10–19	74	37 (50.0)	2.5	1.3	48 (64.9)
20–29	73	36 (49.3)	1.7	2.0	43 (58.9)
30–39	56	35 (62.5)	1.3	1.2	42 (75.0)
40–49	79	49 (62.0)	1.4	1.7	55 (69.6)
50–59	36	18 (50.0)	0.6	1.0	23 (63.9)
60–69	17	7 (41.2)	0.5	0.5	7 (41.2)
70–79	5	3 (60.0)	0.3	0.2	1 (20.0)
80–89	3	3 (100.0)	0.0	0.5	0 (0.0)
Total	421	229 (54.4)	1.2	1.3	248 (59.0)

*Cases/100.000 population, by age category. †Number and proportion of cases for which typing data could be obtained.

MHS determined the most probable modes of transmission for 268 of the 421 reported cases before typing (64%). Travel-associated transmission dominated (141 cases), followed by person-to-person transmission (76), male-to-male sexual contact (33), and foodborne transmission (18) (Table 3).

**Table 3:** Hepatitis A transmission modes, the Netherlands, 2008–2010*

Transmission	No. reported		Sequenced, no. (%)			
			Total	Assigned category confirmed	Unresolved	Assigned category misclassified and reassigned
Travel-associated	141		66 (47)	40 (61)	23 (35)	3 (5)
Person-to-person	76		53 (70)	53 (100)	0	0
Male-with-male sex	33		25 (76)	23 (92)	2 (8)	0
Foodborne	18		17 (94)	7 (41)	9 (53)	1 (6)
Unknown	153		87 (57)	NA	45 (52)	42 (48)
Total	421		248 (59)	123 (50)	79 (32)	46 (19)
*NA, not applicable; if a category was assigned after sequencing, then the assumption “unknown” was misclassified.						

### Sequence Analyses

Of 292 samples received (69% of reported cases) PCR results for HAV were negative for 39 and positive for 253 (5 of which could not be typed and were excluded). The remaining 248 (59% of reported cases) were included in the final analysis. For 21 strains, sequencing was limited to 402–458 nt instead of the goal of 460 nt; for 1 strain, sequencing was limited to 100 nt.

Logistic regression showed that a longer lag time between onset of disease and diagnosis and belonging to the youngest or oldest age groups correlated with negative PCR results for HAV. This finding was expected because of unclear date of disease onset (data not shown).

### Combined Analysis

Typing results confirmed all clusters of suspected person-to-person transmission, nearly all reported cases of male-to-male sexual transmission, and a large proportion of travel-associated infections (Table 3). One third of patients with travel-associated infections had traveled to countries with insufficient HAV sequence information in the public databases for reference. Therefore, the strain sequences for the virus in these patients could not be definitively assigned (category unresolved, Table 3).

In the category of suspected foodborne infections, nearly half of the cases for which sequencing had been performed could be confirmed. Only 1 case was misclassified; this infection was assigned to male-to-male sexual contact because the strain from this patient matched the dominant strain for MSM and the patient’s sexual orientation was concordant with this finding. The remaining cases were considered unresolved because the virus sequences did not cluster with known sequence clusters in the database.

For almost half of the 87 patients with unknown mode of transmission for whom sequencing was performed, the mode of transmission was resolved according to interpretation of the typing results. A remarkably high proportion (52%) of these infections were foodborne (Table 4).

**Table 4:** Hepatitis A virus transmission categories after typing of 42 cases previously assigned to transmission category “unknown,” the Netherlands, 2008–2010

Transmission mode	No. (%)
Travel-associated	2 (5)
Person-to-person	12 (29)
Male-with-male sex	6 (14)
Foodborne	22 (52)
Total	42 (100)


### Probable Foodborne Outbreaks

Cluster 1 began with 2 cases linked to the same restaurant according to notification alone. A cook working in the restaurant had been infected by the dominant strain usually identified in MSM. He had continued working during his illness and was the probable source of infection. After genotyping and additional questioning, 2 more cases were added to this cluster.

Cluster 2 consisted of 2 cases clustered in time. Each patient had a unique genotype IA strain not previously detected, and both patients had eaten mussels.

Clusters 3 and 4 were associated with 2 consecutive outbreaks related to semidried tomatoes (12 and 5 primary cases, respectively). Cluster 3 turned out to be part of the largest foodborne outbreak thus far reported in the Netherlands, reaching 17 cases (including primary and secondary cases). The cases were clustered in time (reported in February and March) but were geographically dispersed, and the national notification rate was at an expected low level for this time of year, according to the 5 previous years. The strain sequences clustered with those from a large outbreak (at least 144 cases) in Australia and an outbreak (59 cases) in France, both of which were associated with consumption of semidried tomatoes (*22*–*24*). Cluster 4 was caused by a genotype IB strain closely resembling the strain involved in cluster 3.

Cluster 5 consisted of 1 case in a food handler of a dinner and 5 secondary cases. Cluster 6 consisted of 5 cases that were clustered strongly in time and for which virus strain sequences were identical, but the cases were geographically dispersed. Although the strain sequences were similar to those of strains typically detected in travelers returning from Morocco, the patients reported no travel history and no contact with patients with HAV infection imported from Morocco. Moreover, they clustered in April, a time of year when secondary or tertiary infections following travel-related imported cases are rare (*8*). Therefore, this cluster was considered a point-source—and very probably foodborne—cluster, although a source could not be determined.

Of 29 foodborne cases confirmed by a combination of epidemiologic and typing information (7 previously suspected foodborne and 22 previously unknown source), 20 additional reports were made. These cases were reported to the national food safety authority and international alerts through the Rapid Alert System for Food and Feed and Early Warning and Response System of the European Commission and the European Centre for Disease Prevention and Control.

### Unresolved Cases

For 45 (52%) cases initially reported as having no known source of infection (Table 3), conclusive evidence for a source was not found despite molecular typing. Nevertheless, some clustering occurred among these unresolved cases. The dominant MSM strain was found in 11 patients; however, these patients were not epidemiologically linked (time, place, food consumption), and among them were women and children, indicating spillover from the MSM risk group to the general population. Several other strains matched background strains previously imported from or known to circulate in Morocco and Egypt and even an outbreak strain from the Czech Republic (*25*). None of these patients had a history of travel. This finding could indicate unnoticed endemic transmission from persons with imported cases, although transmission through food or food handlers could not be excluded.

## Discussion

Use of real-time enhanced molecular surveillance of HAV infections for 2 years enabled us to identify food-associated infections that had not been recognized through regular investigations by MHS. We confirmed almost half of the suspected foodborne cases and resolved a quarter of cases with initially unknown source of infection as probable foodborne infections. Among these infections was an outbreak associated with semidried tomatoes, which was part of an international outbreak. This outbreak would not have been detected without genotyping because baseline surveillance did not generate a signal (*22*). Together, confirmed and unresolved foodborne infections explained 16% of 248 cases for which typing had been performed as opposed to the 4% that had been suspected on the basis of epidemiologic investigation alone. Furthermore, we were able to lower the proportion of cases with unknown mode of transmission from 35% to 18%. On the basis of these findings, we conclude that virus typing is useful for the detection of foodborne outbreaks and, more generally, for the explanation of cases with unknown mode of transmission.

A strength of our study is the representativeness of the study population. In the Netherlands, HAV incidence remained steady at a very low endemic level of ≈200 reported cases per year during 2005–2011 (*26*). Not only did we gather all notification data; we received 69% of patient serum samples. Age distribution was in accordance with the susceptibility of the population of the Netherlands (*27*) and with the distribution described in neighboring Germany (*28*). This study provides a realistic estimation of the incidence of foodborne infection in the Netherlands and maybe in industrialized countries with low HAV endemicity in general, although varying between years with typical epidemic rather than endemic occurrence. The age distribution indicates a risk that food handlers will have an infection and become a source of foodborne infections. Of note, the proportion of foodborne infections was comparable to the proportion of infections among MSM; both types of infection can be epidemic and sporadic.

Real-time investigation of cases enabled us to compare the conclusions that were drawn on interviews alone before typing with those drawn after receiving typing results. The hierarchy of assigning the most probable mode of transmission based on interviews was not standardized, supporting the need for more robust information. Previous studies conducted in industrialized countries have provided insight into nationwide epidemiology supported by molecular typing data (*11*,*28*–*32*), although these studies have not been set up to direct the source tracing. Our study was able to detect foodborne clusters despite the long lag time between infection and notification (average 6 weeks). Routine implementation of standard food-consumption questionnaires at first patient contact and prompt forwarding of serum samples from HAV IgM–positive patients for typing can probably reduce the lag time.

Although we focused on foodborne infections, we have other findings to share. Nearly 100% of suspected cases of person-to-person and male-to-male sexual transmission of HAV could be confirmed; however, additional cases and previously undetected clusters surfaced after sequencing from the category “unknown.” Interventions were altered accordingly, which resulted in 8 additional screening or vaccination actions (data not shown). Only 61% of cases of travel-associated transmission could be confirmed. We have no reason to doubt the patients’ travel history. The most likely explanation is a lack of robust molecular information from many countries. Secondary or additional cases acquired through contact with persons with unnoticed primary cases indeed proved to be part of the explanation for cases with unknown transmission, as expected, although we have shown that this was not the only explanation. In another study, we will aim to combine our data on travel-related risk with data on travel behavior.

A proportion of cases left with unknown source of infection could still have been sporadic foodborne infections or part of undetected international clusters. We are only marginally able to detect such clusters, despite the existence of a shared database provided by GenBank and the early warning networks among public health services and food authorities (Rapid Alert System for Food and Feed and Early Warning and Response System). This marginal ability at least partly results from the fact that typing is often not a structural part of a national surveillance system, and if it is, there is no international consensus on the location and length of the sequenced part of the HAV genome. GenBank offers many more strain sequences for comparison based on shorter sequences (<300 nt) or from different gene fragments, but the robustness of clustering decreases with fragment length (*16*). In addition, metadata in GenBank are often lacking, thereby limiting the usefulness of this repository for molecular epidemiologic studies.

A weakness of our study is that it was not designed to provide estimates for the number of cases prevented. For foodborne outbreaks, altering production processes with risk for contamination or withdrawal of (frozen) products from the market can substantially reduce the number of new cases. The described international outbreak did result in the evaluation of the manufacturing process of semidried tomatoes and a warning published by the food safety authority to inform retailers about risky products. As further illustration of a possible cost benefit, 2 previously healthy persons who were part of a foodborne cluster each needed liver transplantation because of fulminant hepatitis; the costs associated with this treatment alone greatly exceed the costs of 2 years of typing all HAV cases.

A challenge associated with responding to foodborne illness outbreaks is that detection of pathogens in food products typically is requested as support for control activities by a food safety authority. The national food safety authority was not able to confirm any of the suspected foodborne clusters for several reasons but particularly because food leftovers were sparsely available (in part because of the long incubation period), and virus detection in food is challenging (*13*). Nevertheless, contamination of semidried tomatoes with HAV was actually confirmed in the related outbreak in Australia (*23*), and there are examples of HAV infection caused by consumption of food that was contaminated through contact with an infected food handler (*33*) or fecal contamination during food production (e.g., for shellfish or green onions) (*34*–*36*). The largest known outbreak, in Shanghai in 1988, resulted in >250,000 cases linked to the consumption of clams (*37*).

Molecular typing of HAV in patient serum is not routinely performed, and strain typing information is not included in notifications. Combining typing results with anonymized notification data proved to be challenging in our surveillance system. We might have been unable to merge some cases with their typing results because of a lack of unique identifiers, although we believe that this inability to merge cases and typing information occurred randomly and would not have substantially influenced the study results. According to our data, we advise revision of HAV surveillance so that it also provides baseline information to support foodborne illness detection. The revised system should also include mechanisms for rapid exchange of this information internationally, to enhance the ability to detect diffuse outbreaks (*38*). With the fast development and decreasing cost of sequencing technology, routine collection of these types of data will become realistic in the near future and will provide added value for public health work provided such data-sharing mechanisms are developed (*39*). We have recently implemented this recommendation in our national guidelines.

We also recommend that strains uploaded to GenBank be accompanied, at least, by information about time (date of diagnosis or disease onset rather than by date of submission) and space (country where infection most likely was acquired rather than country from which infection was reported). Sufficient molecular background information is needed to be able to notice a distinct cluster. Therefore, broad sampling, data sharing, and centralized interpretation of data should be part of an enhanced surveillance system. The previously described foodborne outbreaks have already proved the usefulness of national and international exchange of epidemiologic and sequence data.
